# Colonization of Urban Habitats: Tawny Owl Abundance Is Conditioned by Urbanization Structure

**DOI:** 10.3390/ani11102954

**Published:** 2021-10-13

**Authors:** Nerea Pagaldai, Juan Arizaga, María V. Jiménez-Franco, Iñigo Zuberogoitia

**Affiliations:** 1 Department of Ornithology, Aranzadi Sciences Society, Zorroagagaina 11, 20014 Donostia-San Sebastian, Spain; jarizaga@aranzadi.eus; 2 Centro de Investigación e Innovación Agroalimentaria y Agroambiental (CIAGRO-UMH), Department of Applied Biology, Miguel Hernández University of Elche, 03202 Elche, Spain; maria.jimenezf@umh.es; 3 Estudios Medioambientales Icarus S.L. C/San Vicente, 8. 6 Planta, Dpto 8. Edificio Albia I., 48001 Bilbao, Spain; zuberogoitia@icarus.es

**Keywords:** binomial *N*-mixture models, landscape metrics, patchy habitat, spatial scales, urban environments, urban raptors

## Abstract

**Simple Summary:**

Alteration of natural habitats due to urbanization is an increasing concern worldwide. Some species, including owls, can exploit this novel environment, although the consequences at the population level have not been described. In this study, we analyze the effect of different urban variables on tawny owl (*Strix aluco*) population abundance. At the local scale, forest and urban cover, as well as the clumpiness index, affected tawny owl abundance. At the landscape scale, its abundance decreased in complex-shaped urban patches and when distance between them was greater. Urban habitats do not substitute natural habitats in terms of abundance, but the species can easily colonize patchy urban habitats.

**Abstract:**

Natural habitats are being altered and destroyed worldwide due to urbanization, leading to a decrease in species abundance and richness. Nevertheless, some species, including tawny owls, have successfully colonized this novel habitat. Consequences at the population level have not been described; thus, our main objective was to describe the effects that urban structure have on the tawny owl population at local and landscape levels. Data were obtained from 527 survey points over 7 months in a large-scale owl survey in the Basque Country (northern Spain) in 2018. At the local scale, the interaction between forest and urban cover affected tawny owl abundance, the optimum being in medium forested areas. The interaction between urban cover and clumpiness index (urban patch distribution) showed a generally negative effect. At the landscape scale, its abundance decreased in complex-shaped urban patches and when distance between them was greater. In conclusion, at the local scale, when a minimal forest structure is present in urbanized areas, the species can exploit it. At the landscape scale, it prefers smaller urban towns to cities. Thinking ahead, the current tendency toward “green capitals” should benefit tawny owl populations.

## 1. Introduction

The process of urbanization has led to the alteration and destruction of the natural habitat and a decrease in species abundance and richness [1]. As a result [2,3], only a few species are able to successfully adapt to this new urban environment. Although habitat loss is a major consequence of the conversion from native to non-native habitats, habitat fragmentation does have distinct and sometimes influential effects (e.g., [4]). The fragmentation of habitats due to the expansion of cities into their urban surroundings affects the habitats available for birds in all cities across the world [5]. However, the ability to cope with the challenges of living in urban areas depends not only on the characteristics of each species but also on local conditions [6]. Despite a large number of negative influences on urban birds [7], cities still allow ecological niches to occur, meaning that those species able to exploit them perform well [8]. While this leads to an increase in avian biomass, the downside is a reduction in richness [9].

Differences have been observed in bird species along rural–urban gradients, with sensitive species generally tending to locate at the outskirts of the city which still have a relatively high proportion of “natural” habitat. However, with increasing expanses of urbanization within the “core” area of the city, the avian community tends to be dominated by four or five “urban” bird species [10]. Raptors are considered “sentinels” of different local and large-scale environmental change, being sensitive to changes in land use and highly susceptible to local extinctions [11]. For instance, raptor richness tends to be negatively affected by urban development [12]. Indeed, urbanization is leading to systematic raptor population declines due to the reduction in the area of suitable habitat available [13,14]. Nevertheless, some studies have revealed that this group of birds has a certain capacity to colonize urban areas [15,16]. This could be due to the fact that raptors usually have large home ranges, which can extend well beyond the urban boundary and, therefore, do not need to meet all their ecological requirements within urban spaces [9]. In addition, moderate levels of landscape alteration may also permit the co-occurrence of many species, mostly due to edge effects [17]. In such conditions, community structure changes along the transition zone, or ecotone, between different land uses [18]. Some generalist species may in fact even show higher densities and reproduction and survival levels in urban areas [19].

Tawny owl (*Strix aluco*) is a ubiquitous and resilient raptor that successfully exploits heterogeneous patchy habitats [20]. Due to its abundance and broad geographic range, it is an appropriate bird species model with which to test how raptors colonize urban environments and which drivers from these urban habitats play a role in explaining the occurrence of these species. It is necessary to understand how urbanization affects the tawny owl at the landscape scale [21,22]. The effect of habitat fragmentation (i.e., forest fragmentation) on tawny owl populations has been studied previously [20,21]; however, to our knowledge, the effect of urban habitat fragmentation remains unknown. In addition, it is not common to study habitat selection through a multiscale approach, even though different variables can be important to a species at different spatial scales. As such, studies conducted at arbitrarily defined scales may suffer from serious limitations [23].

In this work, we aimed to study how the composition of urban areas and their distribution and structure affect tawny owl abundance at two different spatial scales (landscape and local). Various studies [14,24] have shown the negative effect of urbanization on raptors. However, the characteristics that urban habitats need to possess for them to be exploited by tawny owls and whether such characteristics vary at different scales have not been examined. It is, however, known that tawny owls prefer heterogeneous habitats; hence, we might predict that variables such as urban patch distribution (clumpiness index) and the number of urban patches in an area may influence species abundance, although the effect might vary at the local and at the landscape scale. Our specific objectives were to investigate the influence of the quantity, fragmentation, and complexity of urban areas on tawny owl abundance at the landscape and the local scale. In this way, if management measures need to be taken in the future, the data from this work will enable the best scale for the intervention to be selected and provide an understanding of how it will affect tawny owls.

## 2. Materials and Methods

### 2.1. Study Area

**Figure 1:**
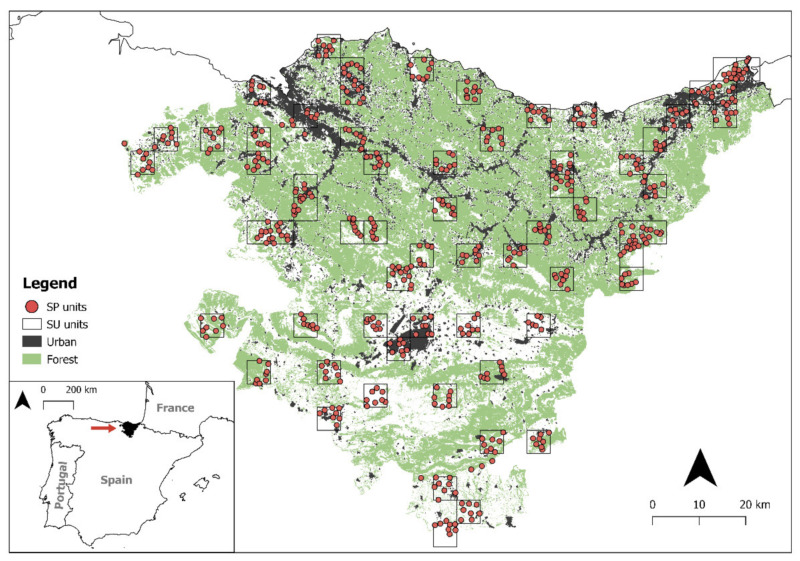
Distribution of the 527 survey points (SPs) and 65 survey units (SUs) in the study area. Forest and urban habitats are shown, which were the land cover types present in the top-ranked model.

This study was carried out in the Basque Country, in northern Spain (7234 km^2^, Figure 1). It comprises two principal biogeographic areas, the Cantabric and the Mediterranean regions, along with a transition zone (subCantabric) between them. The Cantabric region (subprovince Cantabro-Atlantic) lies along the coast of the Bay of Biscay (northern half of the Basque Country). It has an Atlantic climate and mild temperatures (annual mean temperature is 14 °C), and the mean precipitation is 1200–2000 mm. The landscape is mountainous and densely populated, with extensive, dense urban areas located in valleys. Forestry plantations (*Pinus radiata* and *Eucalyptus* spp.) became widespread in the 1980s, gradually replacing grasslands and traditional agricultural activities, but a few remnants of native forest still exist. The Mediterranean region occupies the southern half of the Basque Country. It is drier and has a more continental climate (mean temperature is 20 °C during summer and 4 °C during winter). Mean precipitation is low around the year, approximately 600 mm [24]. The landscape is less mountainous than in the north, being dominated by arable lands, vineyards, Mediterranean scrub, and holm-oak woods, and it is much less densely populated.

### 2.2. Data Collection

The data used in this work were obtained from a large-scale owl survey conducted in 2018 (see details in [25,26]). First, we randomly selected a total of 65 Universal Transverse Mercator (UTM) squares measuring 5 × 5 km that represented all the vegetation types of the Basque Country (Figure 1 and Table 1), each of which was considered to be a survey unit (SU) of 25 km^2^. Second, we selected survey points (SPs) in each SU. These were chosen to represent the main habitat types present in each SU. Each SP was surrounded by a 1 km^2^ buffer zone with a radius 564.2 m, and there was a minimum distance of 1 km between SPs. Overall, we surveyed 527 SP (Table A1). From January to July 2018, we conducted 2507 surveys in total, in the 527 SPs distributed in 65 SUs.

We surveyed owls for seven consecutive months (one survey per month from January to July). As the survey area was very large and the work was carried out over a long period, 24 observers were involved, each of them responsible for surveying a certain number of SUs. In this way, it was possible to have all the SUs surveyed each month, with 4–8 SPs per SU surveyed each time (Table A1; 6.5 ± SD: 0.7; range: 6–8). In each SP, we applied a three-period survey protocol: 5 min listening for spontaneous calls, 5 min of playing recorded calls, and 5 min listening for calls, making a total of 15 min per survey (for more details, see [25,26]). In the recordings, which were created to our own design based on our previous experience (e.g., [27]), we included a mix of territorial and mating calls of both sexes of particular species in tracks lasting 5 min. Calls were downloaded from xeno-canto (https://xeno-canto.org/, accessed on 10 December 2017). The volume of the recording was loud enough to be heard by an observer at a distance of 300 m but not so loud that it caused distortion in the area close to the speakers. All the surveys were conducted during the first few hours after dusk (3.04 ± SD: 1.73). Since surveying eight SPs required on average 180 min, only 1–2 SUs were surveyed in a single night by each observer. Surveys were conducted on dry nights and were suspended if it rained or if it became windy, because these factors significantly reduce owl detectability [25]. The recordings of tawny owl calls were used in February because it is when the species demonstrates high vocal and territorial activity [28,29]. When a tawny owl responded to the recording in any other month, this was also taken into account in the data and analysis, even though the probability of a response was lower. It made it necessary to work with detectability. As a result of this variability in the likelihood of tawny owl responsiveness, the concept of detectability is important to this study. Detectability, or the probability of detection (*p*), is defined as the probability of detecting at least one individual at a specific site during a survey, assuming that individuals are present at the site during the sampling period [30]. Detectability is an important source of variation in monitoring programs and can often account for false negatives. For example, nocturnal species usually have low detectability rates, which means that, even though tawny owls are present, they often do not call and, thus, are not detected [25]. It is, therefore, important to employ a hierarchical modeling framework for tawny owl populations in order to estimate abundance that is corrected for imperfect detection.

### 2.3. Environmental Variables

We selected 13 site-specific covariates (Table 2): (1) REG (categorical), relating to the biogeographic zone (Cantabric, SubCantabric, Mediterranean); (2) ALT (linear), altitude (m a.s.l.) of the SP/SU; (3) ALT^2^ (quadratic), quadratic altitude (m a.s.l.) of the SU. The 527 circular SP buffer areas and 65 square SU areas were overlaid onto digital vegetation and urban maps (www.geoeuskadi.eus, accessed on 15 March 2019) to obtain the percentage of vegetation types; (4) FOR (linear), forested area which incorporated various plantation types; (5) FOR^2^ (quadratic), the quadratic function of the percentage of forested area; (6) URB (linear), percentage of urban area. We also obtained various indices of the urban habitat structure in each SP and SU using the ‘sample_lsm’ function of the ‘landscapemetrics’ package in R. These were as follows: (7) CAI (linear), the core area index, which refers to the central area of the urban patch; (8) CAI^2^ (quadratic), the quadratic effect of CAI for each SU; (9) CLU (linear), the clumpiness index, which refers to the distribution of urban patches, having a value between −1 and 1, and indicates whether the urban area is totally disaggregated (−1), randomly distributed (0), or totally aggregated (1); (10) ENN (linear), the mean Euclidean nearest neighbor, which refers to the distance between the closest two patches having the value of 0 when the nearest neighbor is physically adjoining (aggregated patches) and an unlimited upper limit (isolated patches); (11) SHAPE (linear), the mean shape index of the SP, which is a gradient of perimeter dependent shape complexity and is 0 if all the urban patches are squares, while it increases without limit as the complexity of the patch shape increases; (12) NP (linear), the number of urban patches inside the SP; (13) PAF, the urban perimeter–area fractal dimension for each SU, a value that reflects the complexity of the shape of the SU but is not perimeter dependent and gives a value between 1 and 2, where 1 indicates a shape with a very simple perimeter and 2 indicates a shape with highly convoluted perimeter.

Regarding survey covariates, we used the same ones used in our previously published work surveying the entire owl assemblage [25]. These were (1) BROAD (binomial), whether the individual owl response was before (spontaneous, 0) or during/after the recording (1), (2) EXPER (binomial), level of observer’s experience surveying owls (0 for observers with no previous experience, 1 for those with experience) (see, e.g., [31,32]), (3) DATE (linear), Julian date, used to control for seasonal effects, (4) HOUR (linear), time in the day survey, measured as hours after sunset, and (5) WIND (linear), wind speed (km/h). This variable was obtained from the meteorological station closest to each SP (source: http://www.euskalmet.euskadi.eus, accessed on 15 March 2019).

For the analyses at the local scale (1 km^2^), we took into account the five survey covariates and 10 of the 13 site-specific covariates. CAI^2^ and ALT^2^ were not included because the AIC value increased when adding them into the model. PAF was also dropped because a minimum of 10 patches per SP is needed to measure this index, and most of our SPs did not meet this requirement, although it was taken into account in the analyses at the landscape scale (25 km^2^). The fieldwork at the landscape scale was not designed to consider detectability; therefore, the covariates for the various SPs were not joined as there could be considerable variability from one to another. Thus, in the landscape-scale models, only the 13 site-specific covariates were considered. The survey covariates were not considered (none were included).

### 2.4. Statistical Analysis

The analyses were run at the two spatial scales. The predictive variables recorded for the analysis can be classified into two groups: those that might affect species detectability (survey covariates) and those that might affect tawny owl abundance (site covariates).

We performed binomial *N*-mixture occupancy models where abundance and detectability were estimated as a function of site-specific and survey-specific covariates using the log link function [33]. Observations were generated through a combination of (a) a state process to determine abundance (i.e., counts) at each site, and (b) a detection process that yields observations conditional on the state process [30,34,35]. To control for these two sources of variation, models were built with the “pcount’=” function from the “unmarked” package [33] in R [36]. At the local scale, the abundance of tawny owls per SP was considered as the response variable, with a zero-inflated Poisson distribution. Second, at the landscape scale, the maximum number of tawny owls detected in all the SPs in an SU was considered as the response variable, with a Poisson distribution of errors.

Given the differences between local and landscape scale characteristics, model fitting was conducted in a slightly different way in each case. At the local scale, as the detectability model was previously obtained [24], we kept these variables (DATE, HOUR, WIND, BROAD, and EXPER) constant when performing the abundance model selection, which was performed in a hierarchical process that considered all the possible combinations of the site-specific covariates [36]. We first standardized the variables and fitted both linear and quadratic functions for each variable, and then ran an ANOVA for each pair of models per variable and chose to include the quadratic function when it provided fit better than the linear function. Consequently, we considered the quadratic function of forest (FOR^2^) for the local scale and the quadratic functions of forest (FOR^2^), altitude (ALT^2^), and urban core area index (CAI^2^) for the landscape scale. In addition, in case tawny owl abundance is affected by the interaction of different variables with the urban variable, we tried different saturated models (at both local and landscape scale) with and without interactions. In the case of local scale, the interactions of FOR, FOR^2^, CLU, and NP lowered AIC values; hence, these interactions were included in the model. Model selection was carried out using the AIC criterion [37]. Models differing by <2 AIC units were considered to fit the data equally well, and, in this case, we used model averaging procedures to calculate estimates of regression parameters. The relative importance of each predictor (RVI) was assessed by summing the AIC weights across the highest-ranked models that include that predictor [38].

## 3. Results

Overall, 680 tawny owl calls were noted. Our models showed an evident negative effect of URB on species abundance at both spatial scales (Table 3), although it was strongly influenced by other factors that also impacted species abundance.

### 3.1. Local Scale

**Figure 2:**
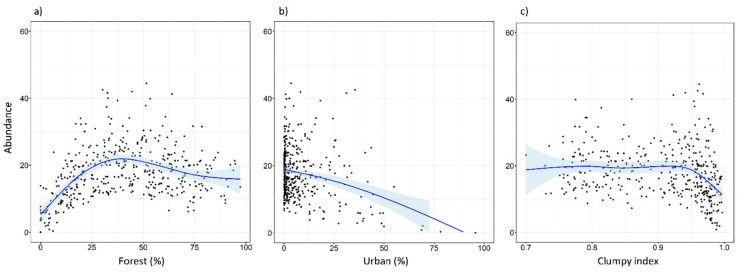
Expected abundance of tawny owl (*Strix aluco*) at the local scale and based on the top-ranked model in relation to (**a**) forested gradient, (**b**) urban gradient, and (**c**) clumpiness index. The clumpiness index represents the aggregation level of habitat patches (in our case, urban patches). It is equal to −1 when the urban patch is maximally disaggregated, 0 when it is randomly distributed, and 1 when maximally aggregated. The blue lines show a cubic smoothing spline fitted to the data in order to show the general trend, while shaded areas show standard error boundaries.

**Figure 3:**
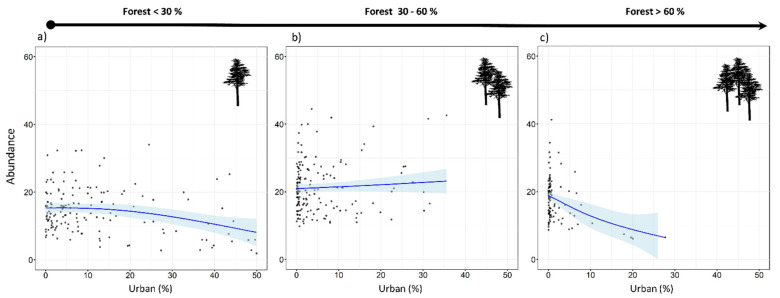
Expected tawny owl (*Strix aluco*) abundance at the local scale and based on the top-ranked model in three forest levels (**a**–**c**) along an urban gradient. The blue lines show a cubic smoothing spline fitted to the data to show the general trend, while shaded areas show standard error boundaries.

**Figure 4:**
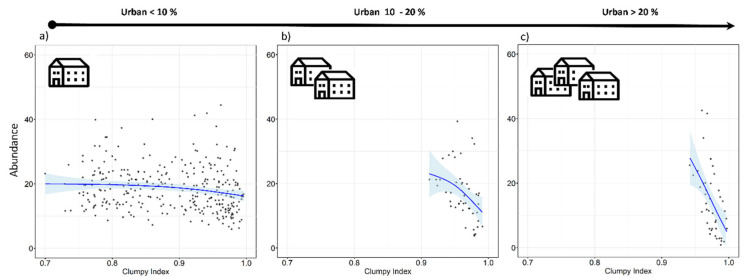
Expected tawny owl (*Strix aluco*) abundance at the local scale and based on the top-ranked model in three levels of urbanization (**a**–**c**) and at different aggregation levels of the urban patches (clumpiness index). The clumpiness index represents the aggregation level of habitat patches (in our case, urban patches). It is equals to −1 when the urban patch is maximally disaggregated, 0 when it is randomly distributed, and 1 when maximally aggregated. The blue lines show a cubic smoothing spline fitted to the data in order to show the general trend, while shaded areas show standard error boundaries.

Tawny owls were detected in 371 SPs (70.4%), at an incidence of 1–5 individuals per SP. Models revealed an effect on abundance for five variables (REG, ALT, FOR^2^, URB, and CLU), as well as the interaction of FOR × URB and CLU × URB (Table 3). These variables were always included as main effects in the top-ranked models (Table A2 and Table A3). There was a positive influence on tawny owl abundance related to Cantabric region, ALT, and FOR (Table 3 and Figure 2). The FOR variable showed a quadratic effect, with the species showing a preference for intermediate percentages of forest cover, rather than highly or poorly forested areas (Figure 2). In contrast, the proportion of urbanized area (URB) showed a negative effect (Figure 2), and CLU revealed tawny owls’ predilection for sites where the urban space is disaggregated (Figure 2). In addition, the FOR × URB interaction showed a negative effect with respect to lowly and highly forested areas (Figure 3) and was optimal in intermediate forested areas, where species abundance was almost constant throughout the urbanization gradient (Figure 3). Models also showed how the effect of urbanization changed depending on its aggregation level (CLU × URB; Figure 4). Urban patch aggregation had almost no effect in lowly urbanized areas but showed a negative effect as urbanization increased (Figure 4).

### 3.2. Landscape Scale

**Figure 5:**
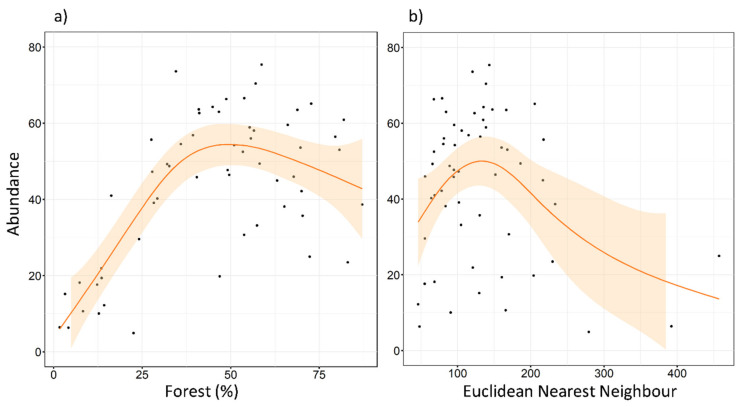
Expected tawny owl (*Strix aluco*) abundance at the landscape scale, based on the top-ranked model along (**a**) a forested gradient, and (**b**) different Euclidean nearest neighbor distances (ENNs) of urban patches. The orange lines show a cubic smoothing spline fitted to the data to show the general trend, while shaded areas show standard error boundaries.

**Figure 6:**
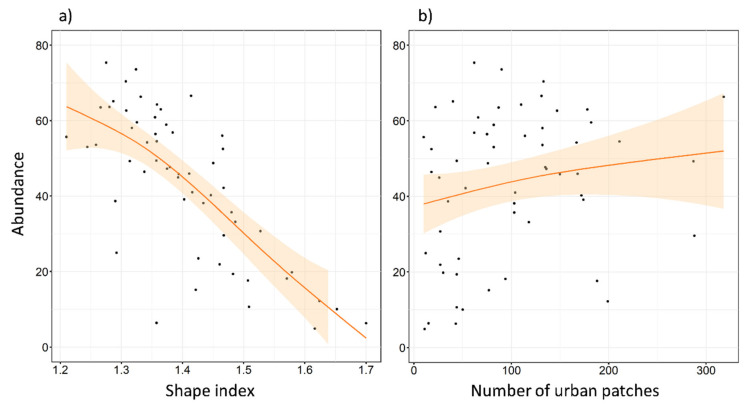
Expected tawny owl (*Strix aluco*) abundance at the landscape scale, based on the top-ranked model with respect to (**a**) different shape index of urban patches, and (**b**) the number of urban patches. The shape index represents the degree of irregularity of habitat patches (in our case, urban habitat). It is equal to 1 when the urban patch is maximally compact (almost square) and increases without limit as the patch shape becomes more irregular. The orange lines show a cubic smoothing spline fitted to the data to show the general trend, while shaded areas show standard error boundaries.

The maximum number of tawny owls detected in a single SU was 22, and the minimum was 0. The mean of the maximum number of individuals detected was 10.49, and the median was 10. Abundance models revealed clear support for five factors (REG, FOR^2^, ENN, SHAPE, and NP), two of them already detected in the local-scale analysis (REG and FOR^2^). No interactions were included at this scale. These factors were always included as the main effect in the top ranked models (Table A2 and Table A3), as well as in the most parsimonious model (Table 3). REG had a very slight effect in favor of the Cantabric region. FOR^2^ showed a negative effect (Figure 5), reaching its highest values at intermediate levels of forested area. ENN and SHAPE also showed a negative effect on species abundance (Figure 5 and Figure 6). Tawny owl abundance decreased when the shape of urban patches was more complex and when the distance between urban patches increased. In contrast, NP had a slight positive effect (Figure 6), with species abundance increasing as the number of urban patches increased.

## 4. Discussion

In a previous study, we analyzed the effects of survey related factors (i.e., the observer’s experience and wind speed) and the principal site-related habitat factors on tawny owl detection and abundance in the study area [25]. We demonstrated that the presence of forested habitats positively affected its abundance, whereas urban habitats had a negative effect. In this study, which complements the previous one, we focused on analyzing the effects the distribution and structure of urban habitats have on the species abundance. Our results show that the abundance of tawny owls in urban habitats strongly depends on the forested areas that surround them, as well as on the structure of the urban area itself. As such, the best sites for tawny owls in cities are periurban areas or areas where an urban–forested matrix is present. Therefore, our results also show new biodiversity potential associated with urban areas as an urban ecosystem service [39] through increasing numbers of tawny owls.

When a local-scale approach was used, the species was found to be more abundant in places with a higher proportion of forests, such as the Cantabric region. This confirms results from our previous study which analyzed the main distribution pattern of this owl in the region [26]. One novel finding, however, is that we detected a quadratic effect, indicating that both extensive forest areas and small patches of forest negatively affected tawny owl abundance (for similar results, see also [40]), this being different to results obtained in another work [41]. It seems, therefore, that the species may benefit from using woodland areas with some degree of forest fragmentation, with the non-forested areas comprising small patches of open habitats such as grasslands, meadows, or pastures [42]. Urbanization showed a forest-dependent effect in addition to its overall negative effect. When the forested area was low (<30%), the percentage of urban area had a slightly negative effect on tawny owl abundance. At intermediate forest percentages (30–60%), urbanization had almost no effect, with abundance being more or less constant throughout the urban range. However, when forest covered extensive areas (>60%), the percentage of urbanized area showed the worst effect. This is probably due to the permanent food supplies that urban environments provide [43], as well as the availability of suitable new nesting and roosting sites [44]. In addition, it has been demonstrated that home range size decreases at the intermediate level of forested area [42], with higher densities being found in urban areas mixed with patchily distributed forests than in purely forested areas [20,45], but always depending on the proportion of suitable habitats available [19]. Regarding the aggregation level of urban patches (CLU), something similar happens in areas of low urbanization (<10%), where aggregation level has a slight, nearly constant, effect on tawny owl abundance. Nevertheless, the negative effect becomes stronger as urbanization level increases, probably due to the lack of suitable habitats since tawny owl is a forest-dwelling raptor and, therefore, needs trees to be able to exploit urban areas. Moreover, in highly urbanized areas (>20%), where urban patches tend to be more aggregated, there are not so many trophic resources to exploit due to the reduced availability of green areas. Tawny owls have been shown to prefer heterogeneous patchy habitats [46], although urban habitat was not taken into account in this study. Our results suggest that this habitat could in fact be included as yet another habitat type that the species can exploit. At this point, we should note that the degree of urbanization within our region ranged from moderate to low since our SUs and SPs were never surrounded by more than 20% of urban land use. This is a good reflection of our study area, which mainly comprises spread out rural villages with few cities.

At the landscape scale, our results also showed that the highest abundance was at intermediate levels of forest cover, again contrasting with previous results [41]. Nevertheless, the amount of urban habitat did not appear to affect abundance, which could be due to the habitat variability encompassed at this scale. However, two urban indices showed significant effects, which means that urbanization affects tawny owl abundance differently when considered at different scales, showing that multiple scales need to be taken into account in future studies [23]. The distance to the nearest neighboring patch of the same class (ENN) resulted in a quadratic effect. That is, when urban patches were very close to each other, the space for forest or crop patches was not big enough or did not contain sufficient diversity to ensure high tawny owl abundances. In contrast, when urban patches were very far apart, the forest or crop habitats between them were larger. As a consequence of tawny owl preference for an urban–rural mix, with high levels of forest or cropland, the abundance of the species will decrease. It is, thus, clear that there is an optimal point where the gap between urban areas is sufficient to maintain a mixture of different crops and forested areas capable of ensuring a high trophic resource, which likely results in an increase in tawny owl abundance. Regarding the urban patch shape index (SHAPE), our results do not match with other researchers [47,48], who observed that the shape index was not relevant to species abundance. In the present study, however, an increase in shape complexity, which is related to a lower urban core area, showed a negative effect on species abundance. This could be attributable to the low urbanization rate in the study area, as well as the narrow range of the variable. The shape index starts at 0 and can increase indefinitely, but our shape index range was only from 1.2 to 1.7. As our shape complexity ratio was so small, we probably did not observe the real effect of this variable. Thus, a wider range of shape complexity should be considered in future studies. The number of urban patches showed a positive effect on species abundance here, which may have been a consequence of the previously mentioned suitable habitats [19] and resource availability, as towns and villages offer high levels of trophic resources and nesting niches.

## 5. Conclusions

Tawny owls have the capacity to colonize new environments [49,50] depending on the surrounding habitat and urban habitat structure [18,20]. This ability is known to be associated with success in the colonization of urban habitats, as well as with ecological and evolutionary consequences in terms of consistent changes in distribution, abundance, behavior, and life history [51]. At the local scale, when minimum forest structure is available in urbanized areas or its surroundings, and when urbanization is patchier, the species can easily exploit it, which has a positive effect on its abundance [18,20]. At the landscape scale, we observed that this species prefers smaller urban villages split in more patches than a large, urbanized city. In this sense, we think that the current tendency toward “green capitals” (the Green Capital Award is a European award for cities based on their environmental records) and the environmental changes involved [52,53] may benefit tawny owl populations, at least in terms of abundance in urban areas. From a biodiversity [54] and pollution [55] point of view, it is valuable to have an urban bioindicator such as an urban tawny owl population, as well as the possibility of monitoring it over time. Nevertheless, we observed that tawny owls do not exploit highly urbanized environments. Our study contributes to the building of friendly cities through adding to our knowledge of how new urban areas should be constructed. It is recommended to maintain small forest patches, transforming the urban matrix into heterogeneous areas, where tawny owls can exploit them as foraging areas in this new urban ecosystem [20]. In the context of global change where landscapes are increasingly anthropized, we want to highlight the importance of assessing multiscale relationships between urban biodiversity and the generation of new urban ecosystem services [39].

## Figures and Tables

**Table 1:** Comparison between the percentage distribution of land types by survey unit (SU, 25 km^2^), by survey point (SP, 1 km^2^), and across the whole study area. Values were obtained by calculating the mean habitat (forest and urban) percentage and standard deviation for each SU and SP area, as well as for the whole study area.

Habitat	SP (Mean % ± SD)	SU (Mean % ± SD)	Study Area (%)
Forest	42.8 ± 26.3	45.1 ± 23.1	54.6
Urban	6 ± 11.7	8.2 ± 11.7	3.1

**Table 2:** Description of the variables used for the top-ranked models at both spatial scales, local and landscape. The “SCALE” column shows the spatial scale used for each variable in the models (LO: local and LA: landscape).

Variables	Contraction	Description	Type	Range	Unit	Scale
Region	REG	Climatic region (Cantabric, Subcantabric, Mediterranean)	Binary	0–1–2	None	LO + LA
Altitude	ALT	Altitude above sea level	Continuous	0–1500	Meters	LO + LA
Forest	FOR	Forested area percentage	Continuous	0–100	%	LO + LA
Urban	URB	Urbanized area percentage	Continuous	0–100	%	LO + LA
Mean core area index	CAI	CAI is the percentage that the core area (interior area) takes in a patch. The mean value of the CAI of all the urban patches in each SP or SU is calculated.	Continuous	0–100	%	LO + LA
Clumpiness index	CLU	Describes how the entire group of urban patches is distributed. Equal to −1 for maximally disaggregated, 0 for randomly distributed, and 1 for maximally aggregated urban patches.	Continuous	−1–1	None	LO + LA
Euclidean nearest mean neighbor	ENN	ENN measures the distance between one urban patch and its nearest urban patch (does not take into account the whole patch group). It is calculated as the mean of ENN of all the urban patches in each SP or SU.	Continuous	>0	Meters	LO + LA
Shape index	SHAPE	SHAPE describes the ratio between the actual perimeter of the urban patch and its hypothetical minimum perimeter. The mean SHAPE value of all urban patches is calculated.	Continuous	≥1	None	LO + LA
Number of patches	NP	The number of urban patches	Discrete	≥1		LO + LA
Perimeter Area Fractal Dimension	PAF	Describes the complexity of an urban patch. Approaches 1 for those with simple shapes (i.e., like a square) and approaches 2 for those that are very irregular.	Continuous	1 ≤ PAF ≤ 2		LA

**Table 3:** Conditional model averaging of the top-ranked models (delta AICc < 2), examining the effect of predicted factors on the abundance of tawny owls. Model average parameter estimates, adjusted standard errors, 95% confidence intervals, and relative variable importance (RVI, the sum of Akaike weights across the set of models in which the variable appears) are shown. Effects with 95% CI not overlapping zero are shown in bold.

Parameters NB	Estimate	SE (Estimate)	Lower 95% CI	Upper 95% CI	RVI
Local scale: 1 km^2^					
**Intercept**	**4.77**	**0.44**	**3.91**	**5.63**	
**REG**	**−0.76**	**0.11**	**−0.98**	**−0.54**	**0.99**
**ALT**	**0.45**	**0.08**	**0.28**	**0.61**	**0.99**
FOR	0.07	0.08	−0.08	0.23	0.99
**FOR^2^**	**−0.25**	**0.08**	**−0.40**	**−0.09**	**0.99**
**URB**	**1.01**	**0.39**	**0.24**	**1.77**	**0.99**
CAI	0.07	0.07	−0.06	0.20	0.89
CLU	−0.51	0.26	−1.01	0.00	0.99
ENN	0.02	0.05	−0.08	0.12	0.83
SHAPE	−0.08	0.06	−0.21	0.05	0.92
NP	−0.08	0.09	−0.25	0.10	0.68
FOR × URB	−0.06	0.16	−0.37	0.25	0.99
**FOR^2^ × URB**	**−0.22**	**0.11**	**−0.44**	**−0.01**	**0.96**
URB × CLU	−0.92	0.48	−1.85	0.01	0.97
URB × NP	0.03	0.06	−0.08	0.14	0.51
Landscape scale: 25 km^2^					
**Intercept**	**3.8**	**1.06**	**1.72**	**5.87**	
REG	−0.14	0.14	−0.42	0.13	0.89
ALT	0.06	0.11	−0.16	0.28	0.90
ALT^2^	−0.13	0.07	−0.26	0.01	0.86
**FOR**	**0.22**	**0.08**	**0.06**	**0.37**	**1**
**FOR^2^**	**−0.21**	**0.08**	**−0.36**	**−0.06**	**1**
URB	0.07	0.1	−0.12	0.26	0.84
CAI	0.31	0.22	−0.12	0.75	0.86
CAI^2^	−0.19	0.13	−0.43	0.06	0.78
CLU	0	0.09	−0.18	0.18	0.80
**ENN**	**−0.6**	**0.22**	**−1.03**	**−0.17**	**1**
PAF	0.08	0.09	−0.11	0.26	1
**SHAPE**	**−0.53**	**0.14**	**−0.8**	**−0.25**	**1**
NP	−0.13	0.08	−0.29	0.03	0.96

## Data Availability

Publicly available datasets were analyzed in this study. This data can be found here: https://doi.org/10.5061/dryad.dncjsxkwg.

## Appendix A

**Table A1:** A summary table of the number of survey points (SPs) in each survey unit (SU) each month. In total, 2507 SPs were surveyed.

(Month)		January	Feburary	March	April	May	June–July	Mean
(Playback Sp.)			*Strix aluco*					Values
SU	Total SPs per SU	SPs Surveyed	SPs Surveyed	SPs Surveyed	SPs Surveyed	SPs Surveyed	SPs Surveyed	SPs Surveyed
30TVN08NW	1	1	0	1	0	0	0	0
30TVN68SE	8	5	6	6	6	8	6	6
30TVN78NW	8	4	6	5	6	7	7	6
30TVN84NW	8	8	8	6	6	8	8	7
30TVN88NW	8	5	8	7	6	6	6	6
30TVN93NW	8	8	8	6	6	8	7	7
30TVN96NE	8	8	8	6	6	6	6	7
30TVN96NW	8	8	7	8	6	6	6	7
30TVN98NW	8	4	6	6	6	7	7	6
30TVN98SW	8	4	8	6	5	6	6	6
30TVN99NW	8	7	4	6	8	6	7	6
30TWN02NE	8	8	8	7	6	6	6	7
30TWN03NE	8	6	8	8	6	8	6	7
30TWN04NW	8	8	8	6	6	6	6	7
30TWN07NW	8	8	7	6	6	6	5	6
30TWN07SW	8	8	8	7	6	6	4	7
30TWN09SW	9	5	5	5	7	6	6	6
30TWN13SE	8	8	8	6	8	8	8	8
30TWN14NE	8	8	8	8	8	8	8	8
30TWN16NE	8	8	8	6	5	5	5	6
30TWN16NW	8	5	7	4	5	8	6	6
30TWN18NW	8	5	6	6	6	7	6	6
30TWN18SE	8	5	6	6	7	6	6	6
30TWN19NW	8	8	8	7	7	7	7	7
30TWN21SE	1	1	1	0	0	0	0	0
30TWN24NE	8	7	5	3	6	6	6	6
30TWN24SW	8	6	6	2	6	6	7	6
30TWN25NW	12	10	9	8	6	4	6	7
30TWN26SE	7	7	7	7	7	7	7	7
30TWN30NE	8	8	8	6	6	6	8	7
30TWN30SW	8	8	8	7	6	7	6	7
30TWN31NE	3	3	3	0	0	0	0	1
30TWN31SW	8	8	5	8	6	6	6	7
30TWN33SW	8	7	6	5	6	5	6	6
30TWN34NE	8	6	8	4	7	8	4	6
30TWN36SE	8	6	6	6	6	6	6	6
30TWN37SW	8	6	6	7	7	8	7	7
30TWN38SW	8	7	7	7	6	8	6	7
30TWN39NE	8	7	7	8	8	7	7	7
30TWN42SW	8	8	8	6	6	6	6	7
30TWN43NW	8	8	4	5	6	6	6	6
30TWN46SE	8	6	6	7	6	7	4	6
30TWN48NW	8	7	7	7	8	8	8	8
30TWN52SW	8	8	8	6	6	6	6	7
30TWN54NW	6	6	6	6	6	6	6	6
30TWN55NE	8	6	6	7	6	6	5	6
30TWN56NW	8	6	6	5	5	6	8	6
30TWN57NE	8	4	6	6	6	6	8	6
30TWN58SE	8	4	6	7	6	6	7	6
30TWN59SW	8	6	4	5	6	6	8	6
30TWN67SW	8	6	5	6	6	7	6	6
30TWN69SW	8	6	5	5	6	6	7	6
30TWN75NW	8	6	6	6	4	7	7	6
30TWN76NE	8	5	7	6	4	7	7	6
30TWN76NW	8	7	5	6	4	7	7	6
30TWN76SW	8	5	7	6	5	7	7	6
30TWN77NE	8	4	6	5	7	7	7	6
30TWN78NE	8	4	6	7	7	7	5	6
30TWN78SW	8	4	6	8	6	6	6	6
30TWN89NE	8	6	5	6	6	6	6	6
30TWN89SW	8	5	7	6	6	6	7	6
30TWN99NW	8	6	5	5	4	7	8	6
30TWN99SW	8	6	4	6	6	7	7	6
30TWP00NE	8	7	7	8	8	8	7	8
30TWP10SW	8	7	7	8	8	8	7	8
30TWP20SE	8	8	7	7	8	8	8	8
30TWP90SE	8	6	4	5	6	8	8	6
30TWP90SW	8	6	4	6	4	7	8	6
**Total**	**527**	422	426	402	399	434	424	

**Table A2:** Local-scale model selection. In the right-hand columns, the AIC, deltaic, and AIC weights (wAIC; used for calculating the RVI index) for each model are shown. The variables used for detectability were as follows: P (DATE + HOUR + WIND + BROAD + EXPER).

Models	Formula	AIC	deltaAIC	wAIC
m.25	REG + ALT + FOR + FOR^2^ + URB + CAI + CLU + ENN + SHAPE + FOR:URB + FOR^2^:URB + CLM:URB	3235.88	0.00	0.28
m.23	REG + ALT + FOR + FOR^2^ + URB + CAI + CLU + SHAPE + NP + FOR:URB + FOR^2^:URB + CLM:URB + NP:URB	3236.91	1.03	0.17
m.29	REG + ALT + FOR + FOR^2^ + URB + CAI + CLU + ENN + SHAPE + NP + FOR:URB + FOR^2^:URB + CLM:URB	3239.94	1.06	0.16
m.21	REG + ALT + FOR + FOR^2^ + URB + CLU + ENN + SHAPE + NP + FOR:URB + FOR^2^:URB + CLM:URB + NP:URB	3237.60	1.73	0.12
m.24	REG + ALT + FOR + FOR^2^ + URB + CAI + CLU + ENN + NP + FOR:URB + FOR^2^:URB + CLM:URB + NP:URB	3238.09	2.21	0.092
m.15 (sat)	REG + ALT + FOR + FOR^2^ + URB + CAI + CLU + ENN + SHAPE + NP + FOR:URB + FOR^2^:URB + CLM:URB + NP:URB	3238.81	2.93	0.064
m.22	REG + ALT + FOR + FOR^2^ + URB + CAI + ENN + SHAPE + NP + FOR:URB + FOR^2^:URB + NP:URB	3239.66	3.78	0.042
m.27	REG + ALT + FOR + FOR^2^ + URB + CAI + CLU + ENN + SHAPE + NP + FOR:URB + CLM:URB + NP:URB	3240.10	4.22	0.034
m.26	REG + ALT + FOR + FOR^2^ + URB + CAI + CLU + ENN + SHAPE + NP + CLM:URB + NP:URB	3241.18	5.30	0.020
m.28	REG + ALT + FOR + FOR^2^ + URB + CAI + CLU + ENN + SHAPE + NP + FOR:URB + FOR^2^:URB + NP:URB	3241.41	5.53	0.018
m.19	REG + ALT + FOR + URB + CAI + CLU + ENN + SHAPE + NP + FOR:URB + CLM:URB + NP:URB	3245.02	9.15	0.003

**Table A3:** Landscape-scale model selection. In the columns on the right, the AIC, deltaAIC, and AIC weights (wAIC; used for calculating the RVI index) for each model are shown. No detectability variables were used at this scale: P(.).

Models	Formula	AIC	deltaAIC	wAIC
mo.11	REG + ALT + ALT^2^ + FOR + FOR^2^ + URB + CAI + CAI^2^ + ENN + PAF + SHAPE + NP	1234.39	0.00	0.22
mo.8	REG + ALT + ALT^2^ + FOR + FOR^2^ + CAI + CAI^2^ + CLU + ENN + PAF + SHAPE + NP	1234.74	0.35	0.18
mo.13	REG + ALT + ALT^2^ + FOR + FOR^2^ + URB + CAI + CAI^2^ + CLU + ENN + SHAPE + NP	1235.55	1.15	0.12
mo.4	REG + FOR + FOR^2^ + URB + CAI + CAI^2^ + CLU + ENN + PAF + SHAPE + NP	1235.81	1.41	0.11
mo.2 (sat)	REG + ALT + ALT^2^ + FOR + FOR^2^ + URB + CAI + CAI^2^ + CLU + ENN + PAF + SHAPE + NP	1236.35	1.96	0.081
mo.9	REG + ALT + ALT^2^ + FOR + FOR^2^ + URB + CLU + ENN + PAF + SHAPE + NP	1236.36	1.97	0.081
mo.3	ALT + ALT^2^ + FOR + FOR^2^ + URB + CAI + CAI^2^ + CLU + ENN + PAF + SHAPE + NP	1236.78	2.39	0.065
mo.5	REG + ALT + FOR + FOR^2^ + URB + CAI + CAI^2^ + CLU + ENN + PAF + SHAPE + NP	1236.81	2.41	0.065
mo.10	REG + ALT + ALT^2^ + FOR + FOR^2^ + URB + CAI + CLU + ENN + PAF + SHAPE + NP	1237.73	3.33	0.041
mo.15	REG + ALT + ALT^2^ + FOR + FOR^2^ + URB + CAI + CAI^2^ + CLU + ENN + PAF + SHAPE	1237.87	3.48	0.038
mo.12	REG + ALT + ALT^2^ + FOR + FOR^2^ + URB + CAI + CAI^2^ + CLU + PAF + SHAPE + NP	1243.04	8.65	0.0029
mo.7	REG + ALT + ALT^2^ + FOR + URB + CAI + CAI^2^ + CLU + ENN + PAF + SHAPE + NP	1243.99	9.60	0.0018
